# The Effectiveness of Respiratory Muscle Training on the Duration and Severity of Respiratory Symptoms in Patients With Chronic Obstructive Pulmonary Disease: A Systematic Review and Meta‐Analysis

**DOI:** 10.1155/carj/6434649

**Published:** 2026-02-06

**Authors:** Zhenghao Yu, Hui Huang, Si Fang, Li Zhang

**Affiliations:** ^1^ Department of Pulmonary and Critical Care Medicine, The Central Hospital of Wuhan, Tongji Medical College, Huazhong University of Science and Technology, Wuhan, Hubei, 443600, China, hust.edu.cn; ^2^ Department of Neurology, The Central Hospital of Wuhan, Tongji Medical College, Huazhong University of Science and Technology, Wuhan, Hubei, 443600, China, hust.edu.cn

**Keywords:** chronic obstructive pulmonary disease, respiratory muscle training, respiratory symptoms, symptom severity

## Abstract

**Objective:**

To evaluate the effectiveness of respiratory muscle training (RMT) on respiratory symptom severity and symptom duration in adults with chronic obstructive pulmonary disease (COPD) and to appraise the certainty of the evidence.

**Methods:**

We searched seven databases and included parallel‐group randomized controlled trials (RCTs) comparing RMT plus guideline‐based care versus control. Meta‐analyses were conducted in RevMan using fixed‐ or random‐effects models as appropriate. Risk of bias was assessed using RoB 2. Certainty of evidence was assessed using the GRADE approach. Because multiple outcomes were analysed, we controlled the false discovery rate using the Benjamini–Hochberg (BH) procedure across prespecified meta‐analysed outcomes and report both unadjusted and BH‐adjusted *p* values.

**Results:**

Seven RCTs (*n* = 1171) were included. No trial reported symptom duration in a way that matched our prespecified definition. Compared with control, RMT reduced dyspnoea (mMRC; unadjusted *p* = 0.003; BH‐adjusted *p* = 0.007; moderate certainty) and improved health‐related quality of life (SGRQ; unadjusted *p* < 0.00001; BH‐adjusted *p* = 3.5e − 05; moderate certainty). Lung function effects were mixed: FVC improved (MD = 0.37 L, 95% CI 0.33 to 0.40; unadjusted *p* < 0.00001; BH‐adjusted *p* = 3.5e − 05; low certainty) and FEV1/FVC improved (MD = 1.84%, 95% CI 0.29 to 3.39; unadjusted *p* = 0.02; BH‐adjusted *p* = 0.035; low certainty), while FEV1 did not differ significantly (MD = 0.18 L, 95% CI −0.04 to 0.40; unadjusted *p* = 0.11; BH‐adjusted *p* = 0.11; low certainty). Evidence for overall symptom burden and exercise capacity was uncertain: CAT (unadjusted *p* = 0.06; BH‐adjusted *p* = 0.07; very low certainty) and 6MWD (unadjusted *p* = 0.05; BH‐adjusted *p* = 0.07; very low certainty).

**Conclusion:**

In adults with COPD, adding RMT to guideline‐based care probably reduces dyspnoea and improves health‐related quality of life (moderate certainty). Evidence for benefits on overall symptom burden, lung function, and exercise capacity is low to very low, and the effect on symptom duration remains unknown due to the lack of reporting.

## 1. Introduction

Chronic obstructive pulmonary disease (COPD) is a common, preventable, and treatable chronic respiratory disease characterized by persistent respiratory symptoms and airflow limitation [1, 2]. In 2019, a modelling analysis based on population‐based spirometry data estimated that about 10% of adults aged 30–79 years—approximately 392 million people—were living with COPD worldwide [1]. According to the Global Burden of Disease (GBD) study, COPD accounted for more than 200 million prevalent cases, 3–3.5 million deaths, and over 70 million disability‐adjusted life years (DALYs) in 2019, making it one of the leading causes of morbidity and mortality globally [3, 4]. COPD also imposes a substantial economic and social burden due to chronic symptoms, frequent exacerbations, loss of productivity, and high healthcare utilization [2, 5].

Current therapeutic strategies for COPD, including smoking cessation, pharmacological bronchodilation, pulmonary rehabilitation, and, when indicated, long‐term oxygen therapy or noninvasive ventilation, are primarily aimed at relieving symptoms, reducing the frequency and severity of exacerbations, improving exercise tolerance and quality of life, and, in some patients, reducing mortality [1, 2, 6] Despite these comprehensive interventions, many patients continue to experience persistent dyspnoea, reduced exercise capacity, and impaired health‐related quality of life, highlighting the need for additional, nonpharmacological approaches that specifically target respiratory symptoms [1, 3, 5].

Respiratory muscle training (RMT) is an important component of pulmonary rehabilitation that aims to improve the strength and/or endurance of inspiratory and/or expiratory muscles through specific training devices or breathing manoeuvres [7–9]. In clinical practice, RMT is usually delivered as simple breathing exercises (such as diaphragmatic breathing and pursed‐lip breathing) and/or as training with mechanical inspiratory muscle trainers that provide a threshold or resistive load against which the patient has to inhale. During these sessions, patients are instructed to breathe more slowly and deeply against the prescribed resistance for a predefined number of breaths or minutes, typically under the guidance of a respiratory therapist or trained nurse at the beginning and then progressively in a home‐based setting. By reducing the respiratory rate, increasing diaphragmatic excursion, and improving the efficiency of respiratory muscles, RMT is expected to decrease the work of breathing and alleviate the perception of dyspnoea in patients with COPD.

Randomized controlled trials (RCTs) and several systematic reviews and meta‐analyses have shown that RMT—particularly inspiratory muscle training (IMT)—can increase maximal inspiratory pressure, enhance exercise capacity, reduce dyspnoea, and improve certain dimensions of health‐related quality of life in patients with stable COPD [10–15]. At present, multiple systematic reviews and meta‐analyses, some of which include more than 30 primary studies, have evaluated the effectiveness of IMT or broader RMT programmes in COPD [10–16]. In line with the concept of “evidence‐based research” proposed by Lund et al., any new systematic review should therefore be explicitly justified by showing how it adds value beyond the existing body of evidence [17, 18].

However, the available reviews also have important limitations. First, most have focused exclusively on IMT delivered with specific threshold devices, whereas clinical pulmonary rehabilitation often combines inspiratory and expiratory muscle training as part of comprehensive RMT programmes [10–13]. Second, previous reviews primarily assessed physiological outcomes (such as maximal inspiratory pressure or generic exercise tests) and did not systematically synthesize data on the duration and severity of respiratory symptoms, even though these are outcomes of direct relevance to patients and clinicians [10–15]. Third, many earlier reviews searched databases only up to around 2016–2018 and rarely included studies published in Chinese‐language databases, which may lead to an under‐representation of more recent trials and of evidence from regions with a high COPD burden [10–15]. According to the evidence‐based research framework, these gaps—uncertainty regarding symptom‐related outcomes, methodological limitations of prior reviews, and the emergence of additional RCTs—justify an updated, focused systematic review [16–18].

Therefore, following the recommendations of Lund and colleagues on evidence‐based research [16], we designed the present systematic review and meta‐analysis to provide an updated synthesis of RMT, used as an adjunct to guideline‐recommended pharmacological therapy and conventional pulmonary rehabilitation, and to evaluate its effectiveness on the duration and severity of respiratory symptoms in patients with COPD.

## 2. Methods

### 2.1. Eligibility Criteria

This systematic review and meta‐analysis was conducted following the methodological guidelines outlined in the *Cochrane Handbook for Systematic Reviews of Interventions* and the *JBI Manual for Evidence Synthesis*. Search reporting adhered to the PRISMA‐S extension, and the study protocol was prospectively registered on the INPLASY platform (registration number: INPLASY202560109).

### 2.2. Inclusion Criteria

Studies were considered eligible for inclusion if they met all of the following criteria:

1. Population enrolled adult participants (≥ 18 years) with a clinical diagnosis of COPD, defined in accordance with contemporary Global Initiative for Chronic Obstructive Lung Disease (GOLD) strategy criteria or equivalent guidelines. Diagnosis required confirmation of persistent respiratory symptoms and postbronchodilator airflow limitation (FEV_1_/FVC ratio < 0.70) by spirometry. All GOLD severity stages (mild to very severe) were eligible. Participants must have been clinically stable, defined as having experienced no acute exacerbation of COPD (i.e., an acute worsening of respiratory symptoms necessitating systemic corticosteroids, antibiotics, or hospitalization) for a minimum of four weeks prior to study randomization.
2. Intervention investigated any structured RMT program. Eligible RMT modalities aimed to improve the strength and/or endurance of inspiratory and/or expiratory muscles, including but not limited to threshold loading devices, resistive breathing training, and targeted respiratory muscle exercises (e.g., diaphragmatic breathing and pursed‐lip breathing). RMT could be delivered as a standalone intervention or as an adjunct to guideline‐based usual care (encompassing both pharmacological and nonpharmacological management).
3. Comparator included a control group (CG) receiving one of the following: (a) usual care (standard management without specific RMT), (b) sham RMT (using devices set at minimal, nontherapeutic load), (c) low‐intensity RMT unlikely to produce a physiological training effect, or (d) another active, non‐RMT intervention.
4. Study design were parallel‐group RCTs. No restrictions were placed on language or publication status during the database searches.

### 2.3. Outcome Measures

The review focused on the following prespecified outcomes:

• Primary outcomes are (a) Severity of respiratory symptoms are patient‐reported symptom burden or dyspnoea measured with validated instruments, including the COPD Assessment Test (CAT), the St. George’s Respiratory Questionnaire (SGRQ; total or symptom domain score), and the modified Medical Research Council (mMRC) dyspnoea scale. (b) Duration of respiratory symptoms is defined a priori as the number of days with respiratory symptoms, the duration of symptomatic episodes, or the time to symptom resolution, as reported in the original trials.
• Secondary outcomes are pulmonary function parameters (forced expiratory volume in one second [FEV_1_], forced vital capacity [FVC], and the FEV_1_/FVC ratio), exercise capacity (assessed by the six‐minute walk distance [6MWD]), and health‐related quality of life (measured by instruments such as the SGRQ).

### 2.4. Exclusion Criteria

Studies were excluded from the review if they met any of the following criteria:

1. Employed nonrandomized or quasirandomized designs, crossover trials without separate analysis of first‐period data, uncontrolled studies, or observational designs.
2. Enrolled mixed patient populations where data specific to participants with COPD could not be distinctly extracted or analysed.
3. Delivered RMT as an integrated, indistinguishable component within a broader, complex pulmonary rehabilitation program, such that its isolated effect could not be determined.
4. Failed to report any data relevant to our prespecified primary or secondary outcomes.
5. Were published only as conference abstracts, letters, editorials, narrative or systematic reviews, meta‐analyses, case reports, or case series.
6. Represented duplicate publications or reported on overlapping datasets (in such cases, only the most complete or most recent report was retained).
7. Presented insufficient or inconsistent outcome data that remained unresolved after reasonable attempts to contact the study authors for clarification.
8. Recruited patients during an acute exacerbation of COPD. An exception was made only for trials where RMT or early rehabilitation was initiated *after* initial clinical stabilization and where outcome data pertaining to that index exacerbation were reported separately from stable‐state outcomes.

### 2.5. Information Sources and Search Strategy

This systematic review was designed and conducted as a comprehensive and reproducible literature search. We searched the following electronic databases from 1 January 2010 to 30 June 2024 without restrictions on publication status: PubMed, EMBASE, the Cochrane Library (including CENTRAL), ScienceDirect, China National Knowledge Infrastructure (CNKI), Chinese Biomedical Database (CBM), VIP database, and Wanfang Data. No language restrictions were applied; search terms were translated into Chinese and adapted to the indexing system of each Chinese database.

The search strategy combined controlled vocabulary (e.g., MeSH terms) and free‐text terms related to COPD and RMT and was developed with reference to the PRISMA‐S statement. For PubMed, the full search strategy was as follows and was last updated on 30 June 2024:

((“Pulmonary Disease, Chronic Obstructive”[Mesh] OR COPD[tiab] OR “chronic obstructive pulmonary disease”[tiab] OR

“chronic obstructive lung disease”[tiab] OR “chronic bronchitis”[tiab] OR emphysema∗[tiab])

AND

(“Respiratory Muscles”[Mesh] OR “respiratory muscle∗”[tiab] OR “inspiratory muscle∗”[tiab] OR “expiratory muscle∗”[tiab] OR

“respiratory muscle training”[tiab] OR “inspiratory muscle training”[tiab] OR “expiratory muscle training”[tiab] OR

“threshold loading”[tiab] OR “breathing exercise∗”[tiab] OR “breathing training”[tiab])

AND

(randomized controlled trial[pt] OR controlled clinical trial[pt] OR random∗[tiab] OR trial[tiab] OR placebo[tiab] OR

“clinical trial[tiab]”))

Equivalent search strategies were constructed for EMBASE and the Cochrane Library using their specific subject headings (e.g. Emtree terms) and syntax. For CNKI, CBM, VIP and Wanfang, we used combinations of Chinese keywords corresponding to COPD (e.g. “慢性阻塞性肺疾病”, “慢性阻塞性肺病”), respiratory muscles (e.g. “呼吸肌”, “吸气肌”, “呼气肌”) and training or exercises (e.g. “呼吸肌训练”, “吸气肌训练”, “呼吸功能训练”), combined with terms for randomized controlled trials. In addition, we manually searched the reference lists of all included trials and relevant systematic reviews to identify additional eligible studies.

The search strategies were adapted appropriately for each database according to its specific indexing terms and search interfaces. In addition, we manually screened the reference lists of all included studies and relevant reviews to identify additional eligible trials.

### 2.6. Study Selection and Data Extraction

All retrieved records were imported into reference management software, and duplicate citations were removed. Two reviewers independently screened the titles and abstracts to identify potentially relevant studies. Full‐text articles were then obtained and assessed independently by the same reviewers against the predefined eligibility criteria. Reasons for exclusion at the full‐text stage were documented. Any disagreements were resolved through discussion or, when necessary, consultation with a third reviewer. The study selection process is illustrated in preferred reporting items for systematic reviews and meta‐analyses (PRISMA) flow diagram.

Two reviewers independently extracted data from each included study using a standardized data extraction form. Extracted information included (1) study characteristics (first author, year of publication, country, setting, and sample size); (2) participant characteristics (age, sex, COPD severity, and inclusion/exclusion criteria); (3) intervention details (type of RMT, training intensity, frequency, duration, and concomitant treatments); (4) comparator details; and (5) outcome data at the end of the intervention and, where available, at follow‐up (pulmonary function, 6MWD, CAT, SGRQ, mMRC, and other symptom‐related outcomes). When required data were missing, unclear, or only graphically presented, we attempted to contact the corresponding authors for clarification or additional information.

For transparency and reproducibility, all extracted study‐level data were compiled into a deidentified dataset, including group‐level sample sizes, means, and standard deviations for continuous outcomes and event counts for dichotomous outcomes for both RMT and control arms at each reported time point. This deidentified dataset forms the basis of all quantitative analyses in this review and is provided as Supporting Table S1.

### 2.7. Risk of Bias Assessment

The risk of bias of the included randomized trials was assessed using RoB 2, the revised Cochrane risk‐of‐bias tool for randomized trials. RoB 2 evaluates bias across five domains: (1) bias arising from the randomization process; (2) bias due to deviations from intended interventions; (3) bias due to missing outcome data; (4) bias in measurement of the outcome; and (5) bias in selection of the reported result. For each domain, signalling questions were answered and the RoB 2 algorithms were applied to reach a judgement of “low risk of bias,” “some concerns,” or “high risk of bias,” and an overall risk‐of‐bias judgement was derived according to the RoB 2 guidance. Two reviewers independently performed the RoB 2 assessments, with discrepancies resolved through discussion or consultation with a third reviewer.

### 2.8. Statistical Analysis

Meta‐analyses were performed using Review Manager (RevMan) version 5.3 (Cochrane Collaboration). For dichotomous outcomes, odds ratios (ORs) with 95% confidence intervals (CIs) were calculated. For continuous outcomes, weighted mean differences (WMDs) with 95% CIs were calculated when outcomes were measured on the same scale; when different scales were used, standardized mean differences (SMDs) with 95% CIs were used. When studies reported change‐from‐baseline data, these were preferred; otherwise, postintervention values were extracted. Because multiple outcomes were analysed, we controlled the false discovery rate using the Benjamini–Hochberg (BH) procedure across the prespecified meta‐analysed outcomes and report both unadjusted and BH‐adjusted *p* values; interpretation focuses on BH‐adjusted *p* values.

Statistical heterogeneity among studies was assessed using the chi‐squared test (with *p* < 0.10 indicating statistically significant heterogeneity) and quantified using the *I* ^2^ statistic. When heterogeneity was low (*I* ^2^ < 50%), a fixed‐effect model was applied; when heterogeneity was substantial (*I* ^2^ ≥ 50%), a random‐effects model was used if pooling of data was considered appropriate based on clinical and methodological similarity. When heterogeneity could not be adequately explained or data were insufficient for pooling, a narrative synthesis was performed instead of meta‐analysis.

Publication bias was planned to be assessed using funnel plots and Egger’s test when at least 10 studies were available for a given outcome. However, because fewer than 10 trials were included in each meta‐analysis, formal assessment of publication bias was not conducted.

### 2.9. Certainty of the Evidence

The certainty of the body of evidence for key outcomes (respiratory symptom severity measured by CAT, mMRC, and SGRQ; pulmonary function indices; 6MWD; and health‐related quality of life) was assessed using the Grading of Recommendations Assessment, Development, and Evaluation (GRADE) approach. For each outcome, the certainty of evidence started as “high” (because all included studies were RCTs) and could be downgraded by one or two levels based on the following domains: risk of bias, inconsistency, indirectness, imprecision, and publication bias. When applicable, the certainty could be upgraded for factors such as a large magnitude of effect or evidence of a dose–response relationship.

Summary of findings tables was prepared using GRADEpro GDT software, presenting the effect estimates and the GRADE certainty ratings (high, moderate, low, or very low) for each outcome to support interpretation of the results and formulation of conclusions.

## 3. Results

### 3.1. The Outcomes of the Literature Search

**FIGURE 1:**
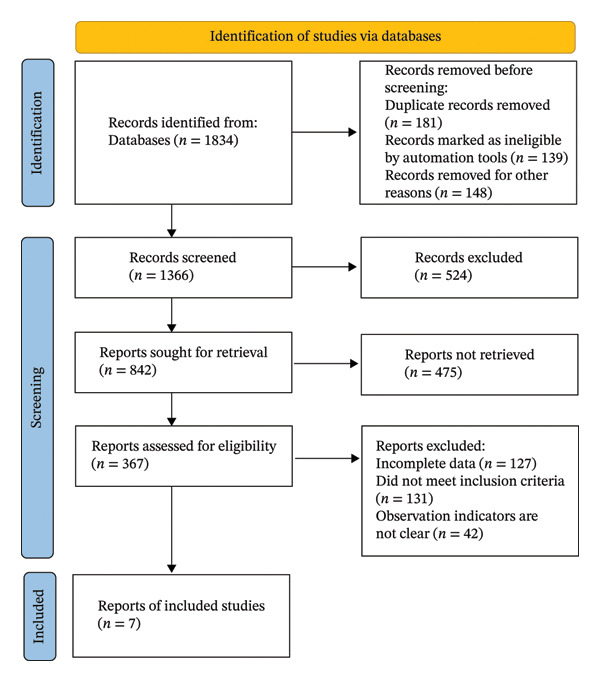
Flowchart of literature screening.

The literature review was carried out in accordance with the PRISMA guidelines. The database search covered the period from 1 January 2010 to 30 June 2024. Initially, 1834 entries were found via searches in electronic databases. After removing duplicates, 1366 studies remained. 842 papers were chosen for additional review after a preliminary screening of abstracts and titles. Following the exclusion of irrelevant studies, reviews, case reports, and nonrandomized studies, 367 articles were deemed potentially eligible. Full‐text screening was then performed, during which 360 studies were disregarded because they lacked key outcome measures or had insufficient data. In the end, the meta‐analysis contained 7 RCTs with 1171 people overall. Figure 1 displays the literature screening flowchart. The main characteristics of the seven included RCTs [19–25] are presented in Table 1, including the first author, year of publication, country, sample size, COPD severity, type and duration of RMT, comparator, and main outcome measures. Deidentified study‐level outcome data extracted from each trial [19–25], including group‐level means, standard deviations, and event counts for all prespecified outcomes, are reported in Supporting Table S1 to allow independent verification and reanalysis.

**TABLE 1:** Basic characteristics of the included randomized controlled trials.

Study (first author, ref.)	Publication year	Country/region	Sample size (*n*)	Intervention (RMT programme)	Control intervention	Outcome indicators^∗^
Jiang et al. [19]	2023	China	84	Early pulmonary rehabilitation training, mainly including pursed‐lip breathing, deep breathing exercises, peripheral muscle strength training, sitting breathing, and multidirectional breathing training	Routine nursing	①②③
Wu [20]	2020	China	17	Drug treatment combined with pursed‐lip breathing and abdominal breathing training	Drug treatment alone	①②③④⑤
Cheng and Luo [21]	2023	China	160	Respiratory muscle function exercises combined with deep‐breathing gymnastics	Conventional nursing model	①②⑤
Xiang [22]	2025	China	150	Diaphragmatic breathing, pursed‐lip breathing, and lung expansion exercises	Routine nursing	①③⑤
Wang et al. [23]	2020	China	124	Conventional Western medicine–based treatment combined with respiratory muscle function training	Conventional Western medicine–based treatment	①②④
Beaumont et al. [24]	2015	Europe (France)	34	Inspiratory muscle training performed with an inspiratory muscle trainer during pulmonary rehabilitation	Sham (false) inspiratory muscle training	②
Schultz et al. [25]	2018	Europe	602	High‐intensity inspiratory muscle training during a 3‐week inpatient pulmonary rehabilitation programme	Sham inspiratory muscle training	①②③④

*Note:* ① Pulmonary function indices (FEV1, FVC, and FEV1/FVC). ② 6‐minute walk distance (6MWD). ③ St. George’s Respiratory Questionnaire (SGRQ) score. ④ COPD Assessment Test (CAT) score. ⑤ Modified Medical Research Council (mMRC) dyspnoea score.

^∗^Outcome indicators.

In addition to the forest plots, the pooled treatment effects are summarized in Table 2, which reports, for each outcome, the number of trials and participants contributing data and the corresponding pooled estimates (mean difference for continuous variables and OR for dichotomous outcomes) with 95% CIs. Because several of the included studies [19–25] did not report all outcomes of interest or did not provide sufficient numerical data (e.g., standard deviations or exact event counts), the total number of trials contributing to each pooled estimate is smaller than the overall number of included RCTs.

**TABLE 2:** Summary of pooled effects of respiratory muscle training versus control.

Outcome	No. of RCTs	Effect measure^∗^	Pooled effect (95% CI)	*p* value (unadj; BH)	Note
FEV_1_ (L)	4	MD	0.18 (−0.04–0.40)	0.11; 0.11	Postintervention values
FVC (L)	3	MD	0.37 (0.33–0.40)	< 0.00001; 3.5e − 05	Postintervention values
FEV_1_/FVC (%)	4	MD	1.84 (0.29–3.39)	0.02; 0.035	Postintervention values
6‐minute walk distance (6MWD, m)	6	MD (NR)	NR (not reported in text)	0.05; 0.07	Pooled MD and 95% CI are shown in the forest plot (Figure 2).
SGRQ total score	3	MD/SMD (NR)	NR (not reported in text)	< 0.00001; 3.5e − 05	Pooled effect size and 95% CI are shown in the forest plot (Figure 3).
CAT score	3	MD/SMD (NR)	NR (not reported in text)	0.06; 0.07	Pooled effect size and 95% CI are shown in the forest plot (Figure 4).
mMRC dyspnoea score	3	MD/SMD (NR)	NR (not reported in text)	0.003; 0.007	Pooled effect size and 95% CI are shown in the forest plot (Figure 5).

^∗^For outcomes measured on the same scale, weighted mean differences (MD) with 95% CIs were calculated; for those measured on different scales, standardized mean differences (SMDs) with 95% CIs were applied.

**FIGURE 2:**
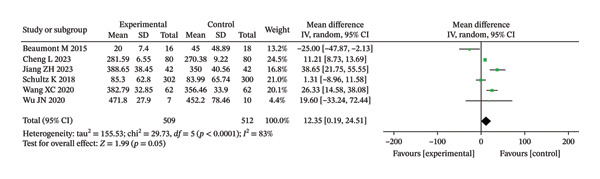
Forest analysis diagrams comparing 6MWD between the two groups after intervention.

**FIGURE 3:**
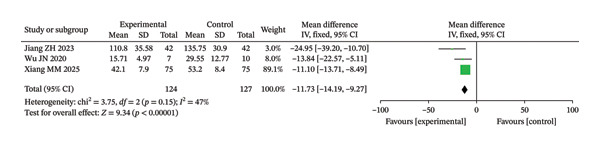
Forest analysis chart comparing the SGRQ scores of the two groups after intervention.

**FIGURE 4:**
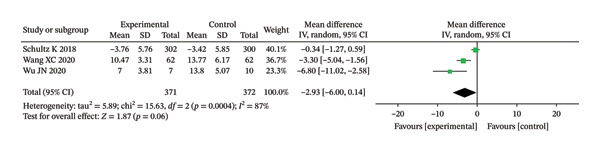
Forest analysis chart comparing CAT scores of the two groups after intervention.

**FIGURE 5:**
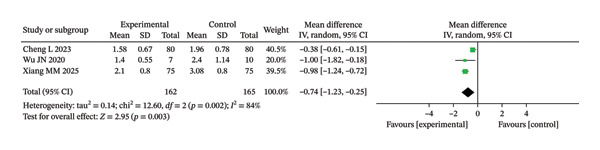
Forest analysis chart comparing the mMRC scores of the two groups after intervention.

### 3.2. Assessment of the Methodological Quality of the Literature

**FIGURE 6:**
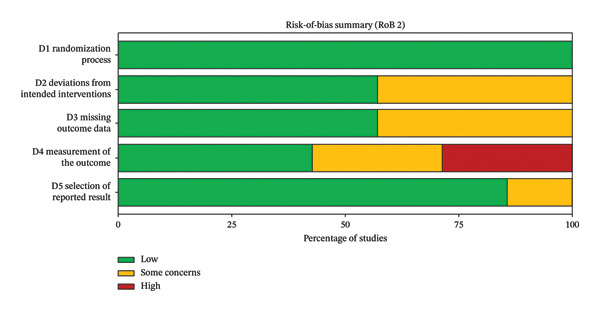
Risk‐of‐bias summary of included trials (RoB 2).

**FIGURE 7:**
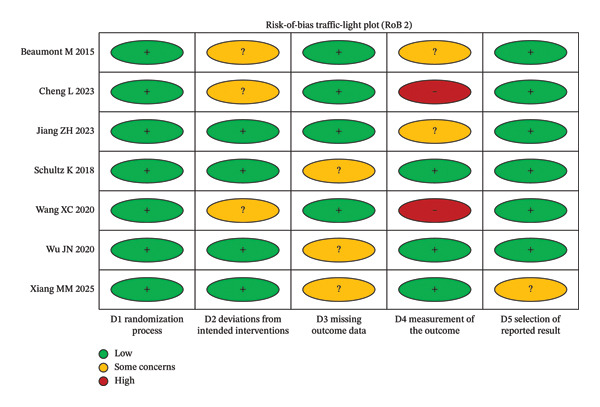
Risk‐of‐bias traffic‐light plot by RoB 2 domain for included trials.

All seven included RCTs reported baseline characteristics and described the intervention procedures and outcome measures. Using the RoB 2 tool, most trials were judged as having “some concerns,” mainly because of insufficient information about the randomization process and/or potential bias due to deviations from intended interventions (e.g., lack of blinding) and, in some cases, concerns about measurement of the outcome (e.g., unblinded outcome assessment) (Figures 6 and 7).

### 3.3. Meta‐Analysis Results

None of the seven included RCTs reported quantitative data that matched our predefined definition of duration of respiratory symptoms; therefore, no quantitative synthesis was possible for this primary outcome, and the results below focus on symptom severity and secondary outcomes.

#### 3.3.1. Respiratory Symptom Severity

##### 3.3.1.1. CAT

In this study, three literatures compared the CAT scores of the two groupings after intervention. The meta‐analysis results indicated significant HET among the included studies (Chi^2^ = 15.63, df = 2, *p* = 0.0004, *I* ^2^ = 87%). Therefore, a REM was used for the analysis (Figure 4). Although the IG showed a greater improvement in respiratory symptoms compared to the CG, there was no discernible change (unadjusted *p* = 0.06; BH‐adjusted *p* = 0.07).

##### 3.3.1.2. mMRC

In this study, three literatures compared the scores of the mMRC scale after two groups of interventions. The meta‐analysis results revealed significant HET among the included studies (Chi^2^ = 12.60, df = 2, *p* = 0.002, *I* ^2^ = 84%). Therefore, a REM was applied for the analysis (Figure 5). The results revealed that the degree of dyspnoea in the IG was considerably reduced compared to the CG (unadjusted *p* = 0.003; BH‐adjusted *p* = 0.007). No dichotomous outcomes that met our predefined criteria were consistently reported across trials; therefore, no meta‐analysis of dichotomous variables or formal assessment of publication bias (e.g., funnel plots) could be performed.

##### 3.3.1.3. SGRQ

In this study, three RCTs reported postintervention SGRQ scores. The meta‐analysis results showed no significant HET among the included studies (Chi^2^ = 3.75, df = 2, *p* = 0.15, *I* ^2^ = 47%). Therefore, the fixed‐effect model was used (Figure 3). The findings demonstrated that the IG revealed considerably greater improvement in quality of life (unadjusted *p* < 0.00001; BH‐adjusted *p* = 3.5e − 05).

#### 3.3.2. Pulmonary Function

##### 3.3.2.1. FEV_1_

**FIGURE 8:**
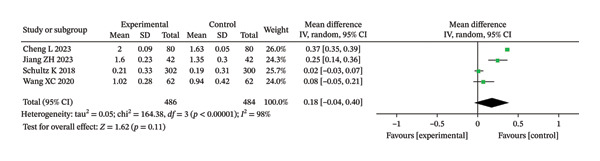
Forest analysis diagrams comparing FVE1 between the two groups after intervention.

Among the seven included RCTs, four studies reported postintervention FEV_1_. The heterogeneity test revealed substantial variability across studies (Chi^2^ = 164.38, df = 3, *p* < 0.00001, *I* ^2^ = 98%). Therefore, a random‐effects model was applied for the meta‐analysis. As shown in Figure 8, there was no statistically significant difference in postintervention FEV_1_ between the intervention group (IG) and the CG following RMT (MD = 0.18 L; 95% CI: −0.04 to 0.40; unadjusted *p* = 0.11; BH‐adjusted *p* = 0.11).

##### 3.3.2.2. Forced Vital Capacity

**FIGURE 9:**
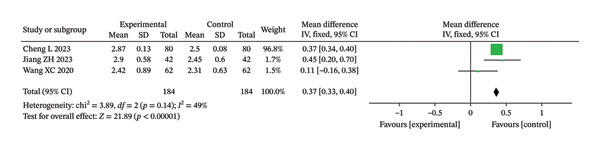
Forest analysis diagrams comparing FVC after intervention in the two groups.

In this study, three RCTs reported postintervention FVC values. The meta‐analysis showed low to moderate heterogeneity among the included studies (Chi^2^ = 3.89, df = 2, *p* = 0.14, *I* ^2^ = 49%). Therefore, a fixed‐effect model was applied for data synthesis (Figure 9). The analysis indicated that the improvement in FVC was significantly greater in the IG compared with the CG (MD = 0.37 L; 95% CI: 0.33–0.40; unadjusted *p* < 0.00001; BH‐adjusted *p* = 3.5e − 05).

##### 3.3.2.3. FVE_1_/FVC

**FIGURE 10:**
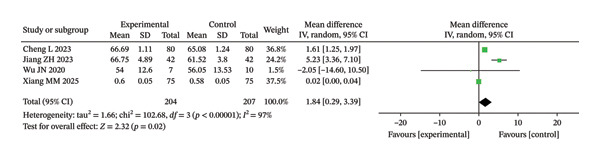
Forest analysis diagrams comparing FVE1/FVC after intervention in the two groups.

In this study, four RCTs reported postintervention FEV_1_/FVC ratios. The meta‐analysis demonstrated considerable heterogeneity among the included studies (Chi^2^ = 1.84, df = 3, *p* < 0.00001, *I* ^2^ = 97%). Therefore, a random‐effects model was used for the analysis (Figure 10). The findings indicated that, compared with the CG, the IG showed a greater improvement in the FEV_1_/FVC ratio after RMT (MD = 1.84%; 95% CI: 0.29–3.39). This effect was statistically significant before multiplicity correction (unadjusted *p* = 0.02) and remained statistically significant after BH FDR correction (BH‐adjusted *p* = 0.035).

#### 3.3.3. 6MWD

The meta‐analysis of the 6MWD suggested a small increase in walking distance with RMT compared with controls, but the pooled effect did not reach statistical significance (unadjusted *p* = 0.05; BH‐adjusted *p* = 0.07) (Figure 2).

### 3.4. Certainty of the Evidence (GRADE)

According to the GRADE assessment, the certainty of evidence was rated as moderate for SGRQ total score and mMRC dyspnoea (downgraded one level for serious risk of bias). Certainty was low for FEV_1_, FVC, and FEV_1_/FVC (downgraded for serious risk of bias and serious inconsistency) and very low for CAT score and 6MWD (downgraded for serious risk of bias, serious inconsistency, and serious imprecision; CAT was also downgraded for serious indirectness). The reasons for downgrading for each outcome are provided in the summary of findings table (Supporting Table S2).

## 4. Analysis and Discussion

In the field of respiratory medicine, COPD is a highly prevalent condition for which pharmacological interventions alone often prove inadequate in fully managing symptoms and functional impairment. Beyond conventional pharmacotherapy, nonpharmacological approaches—especially pulmonary rehabilitation—have become essential components of holistic COPD management. Pulmonary rehabilitation integrates individualized exercise training, RMT, patient education, and self‐management strategies, with the primary goals of improving exercise tolerance, alleviating dyspnoea, and enhancing health‐related quality of life. RMT specifically focuses on strengthening the diaphragm and other inspiratory and expiratory muscles through techniques such as diaphragmatic breathing, pursed‐lip breathing, deep‐breathing exercises, and inspiratory muscle loading (resistive or threshold‐based). By promoting slower, deeper, and more coordinated breathing patterns, these interventions improve diaphragmatic excursion, reduce dynamic hyperinflation, decrease the work of breathing, and delay the onset of respiratory muscle fatigue. For clinicians less familiar with RMT, it is worth noting that these techniques are generally simple, noninvasive, easily integrated into routine rehabilitation or nursing care, and well tolerated by most COPD patients.

A prior meta‐analysis by Ammous et al. [26] demonstrated the efficacy of IMT in COPD management and emphasized that combining IMT with comprehensive PR tends to yield more substantial and consistent clinical benefits. In line with these findings, the present study observed a clear improvement in FVC in the IG; however, the apparent improvement in the FEV_1_/FVC ratio did not remain statistically significant after BH FDR correction, suggesting that RMT enhances respiratory muscle strength and coordination, thereby optimizing alveolar ventilation and mitigating dynamic pulmonary hyperinflation. In contrast, RMT did not produce a statistically significant improvement in FEV_1_ (MD = 0.18, 95% CI: −0.04 to 0.40, *p* = 0.11), a result consistent with earlier evidence [27]. This suggests that the benefits of RMT are mediated primarily through respiratory mechanical optimization rather than structural reversal of airway pathology. As a key marker of airflow obstruction, FEV_1_ tends to respond slowly to intervention and is influenced by numerous physiological and pathological variables. The observed lack of significant change may reflect the need for longer or more intensive training protocols, or it may indicate that FEV_1_ is not the most sensitive outcome for capturing the functional improvements associated with RMT. The considerable heterogeneity across studies (I^2^ = 98%) further underscores the influence of variability in training protocols, intervention duration, and patient characteristics.

Regarding exercise capacity, our meta‐analysis indicated that patients receiving RMT walked a greater distance in the 6MWD compared to controls, though this difference did not reach statistical significance (unadjusted *p* = 0.05; BH‐adjusted *p* = 0.15). This suggests a potential positive effect of RMT on exercise tolerance in COPD, though its clinical meaningfulness remains uncertain. Improvements in exercise capacity are multifactorial, influenced not only by respiratory muscle function but also by cardiovascular fitness, peripheral muscle strength, and overall physical conditioning [28].

In terms of symptom burden, the IG showed greater improvement in CAT scores relative to controls, though again without statistical significance (unadjusted *p* = 0.06; BH‐adjusted *p* = 0.15). Significant heterogeneity was noted across these studies (*χ* ^2^ = 15.63, *I* ^2^ = 87%), which may reflect the multidimensional nature of the CAT—encompassing cough, sputum, and dyspnoea—whereas RMT primarily targets dyspnoea relief rather than cough or sputum production [29]. Accordingly, when dyspnoea was assessed specifically using the mMRC scale, a statistically significant reduction was observed in the RMT group (unadjusted *p* = 0.003; BH‐adjusted *p* = 0.015), consistent with the findings of Buran Cirak et al. [30]. This underscores the specific role of RMT in alleviating breathlessness, a core symptom that profoundly affects daily function and quality of life.

Finally, health‐related quality of life—assessed via the SGRQ—showed significant and consistent improvement with RMT (unadjusted *p* < 0.00001; BH‐adjusted *p* < 0.0001), with low heterogeneity across studies (*I* ^2^ = 47%). These results align with prior reports [31] and reinforce the value of RMT as a key element of pulmonary rehabilitation, capable of enhancing patients’ perceived well‐being and social participation through reduced dyspnoea and improved functional capacity. Together, these findings highlight that while RMT may not uniformly affect all COPD‐related outcomes, it delivers meaningful benefits in respiratory mechanics, symptom‐specific relief, and quality of life, particularly when embedded within a comprehensive, individualized rehabilitation framework.

From a clinical perspective, RMT should not be considered a “novel” stand‐alone alternative to established COPD treatments, but rather a nonpharmacological adjunct added on top of guideline‐based pharmacological therapy and conventional pulmonary rehabilitation. The novelty of the present review lies in its broader temporal coverage and patient‐centred focus: compared with earlier systematic reviews that mainly included trials published up to 2016–2018 and emphasized physiological outcomes, our analysis incorporates more recent trials (including several Chinese‐language RCTs conducted after 2019) and evaluates symptom severity, dyspnoea, and health‐related quality of life as key outcomes.

This study has several limitations. First, only seven RCTs were included in this review. Although we searched multiple international and Chinese databases over a long time frame and did not apply language restrictions, relatively few trials met our prespecified eligibility criteria. Many potentially relevant reports were excluded because they were not randomized, combined RMT with broader pulmonary rehabilitation programmes without separately reporting the effects of RMT, or did not provide data on our primary outcomes related to the severity or duration of respiratory symptoms (e.g., CAT, SGRQ, mMRC, or symptom duration measures). As a consequence, the total sample size is modest, which reduces the statistical power of the meta‐analyses, increases the imprecision of the pooled estimates, and limits our ability to conduct more detailed subgroup or publication bias analyses. One of the included trials evaluated early pulmonary rehabilitation during an acute exacerbation, which may limit the generalizability of our findings to stable COPD populations.

Second, some of the included studies had methodological limitations, such as the absence of blinding procedures and incomplete reporting of loss to follow‐up, which may introduce a risk of bias.

Third, substantial heterogeneity was observed for several outcome measures, including FEV_1_/FVC, CAT score, and mMRC score. This heterogeneity may be related to variability across studies in patient characteristics, intervention content, training intensity and duration, and concomitant treatments, all of which can influence the observed effects of RMT.

Finally, the lack of standardized and validated tools for assessing the *duration* of respiratory symptoms limited our ability to perform an in‐depth analysis of this outcome dimension.

## 5. Conclusion

This review suggests that RMT added to guideline‐based care probably reduces dyspnoea and improves health‐related quality of life (moderate‐certainty evidence). However, evidence for benefits on overall symptom burden, lung function, and exercise capacity is low to very low, and no included trial reported data on the duration of respiratory symptoms as predefined. Future trials should be adequately powered, use standardized symptom outcomes (including symptom duration), and report methods and results transparently to strengthen the certainty of the evidence.

## Funding

The authors received no financial support for the research, authorship, and/or publication of this article.

## Disclosure

This study protocol has been previously registered on the INPLASY platform (registration number: INPLASY202560109).

## Ethics Statement

The authors have nothing to report.

## Conflicts of Interest

The authors declare no conflicts of interest.

## Supporting Information

Supporting Table S1: Deidentified study‐level data from included randomized controlled trials.

Supporting Table S2: GRADE evidence profile for main outcomes (reasons for downgrading are provided below the table).

## Supporting information


**Supporting Information** Additional supporting information can be found online in the Supporting Information section.

## Data Availability

The data that support the findings of this study are available from the corresponding author upon reasonable request. All deidentified data extracted from the included randomized controlled trials and used in the present analyses are provided in Supporting Table S1. Any additional information related to the data extraction process is available from the corresponding author upon reasonable request.
