# Supplementary material for: The Effectiveness of Respiratory Muscle Training on the Duration and Severity of Respiratory Symptoms in Patients With Chronic Obstructive Pulmonary Disease: A Systematic Review and Meta‐Analysis
Source: Can Respir J. 2026 Feb 6;2026:6434649. doi: 10.1155/carj/6434649 (PMC12878794; doi:10.1155/carj/6434649)
Supplement: Supplementary file 1 — Supporting Information Additional supporting information can be found online in the Supporting Information section. [file CARJ-2026-6434649-s001.docx]

Supplementary Table S1. De-identified study-level data from included randomized controlled trials

| **Study (first author, year)** | **Follow-up duration** | **Group** | **n** | **FEV1 (L)** | **FVC (L)** | **FEV1/FVC (%)** | **6MWD (m)** | **SGRQ total score** | **CAT score** | **mMRC score** |
| --- | --- | --- | --- | --- | --- | --- | --- | --- | --- | --- |
| Jiang 2023^19^ | 2 months | Early pulmonary rehabilitation group | 42 | 1.60 ± 0.23 | 2.90 ± 0.58 | 66.75 ± 4.89 | 388.65 ± 38.45 | 110.80 ± 35.58 | NR | NR |
| Jiang 2023^19^ | 2 months | Control group | 42 | 1.35 ± 0.30 | 2.45 ± 0.60 | 61.52 ± 3.80 | 350.00 ± 40.56 | 135.75 ± 30.90 | NR | NR |
| Wu 2020 (stepwise RMT, master's thesis)^20^ | 12 weeks | Control group (drug therapy only) | 10 | NR | NR | NR | NR | 29.55 ± 12.77 | NR | NR |
| Wu 2020 (stepwise RMT, master's thesis)^20^ | 12 weeks | Pursed-lip + abdominal breathing group | 9 | NR | NR | NR | NR | 15.71 ± 4.97 | NR | NR |
| Wu 2020 (stepwise RMT, master's thesis)^20^ | 12 weeks | Breathing trainer group | 7 | NR | NR | NR | NR | 24.03 ± 14.27 | NR | NR |
| Cheng 2023^21^ | 6 months | Respiratory muscle training + deep-breathing exercises group | 80 | 2.00 ± 0.09 | 2.87 ± 0.13 | 69.69 ± 1.11 | 281.59 ± 6.55 | NR | NR | 1.58 ± 0.67 |
| Cheng 2023^21^ | 6 months | Routine nursing group | 80 | 1.63 ± 0.05 | 2.50 ± 0.08 | 65.08 ± 1.24 | 270.38 ± 9.22 | NR | NR | 1.96 ± 0.78 |
| Xiang 2025 ^22^ | 12 weeks | Standardized breathing-training group | 75 | NR | NR | 0.60 ± 0.05 | NR | 42.1 ± 7.9 | NR | 2.1 ± 0.8 |
| Xiang 2025^22^ | 12 weeks | Control group | 75 | NR | NR | 0.58 ± 0.05 | NR | 53.2 ± 8.4 | NR | 3.0 ± 0.8 |
| Wang 2020 | 3 months | Respiratory muscle-function training group | 62 | 1.02 ± 0.28 | 2.42 ± 0.89 | FEV1% 41.30 ± 12.23 | 382.79 ± 32.85 | NR | 10.47 ± 3.31 | NR |
| Wang 2020 | 3 months | Control group | 62 | 0.94 ± 0.42 | 2.31 ± 0.63 | FEV1% 39.88 ± 12.40 | 356.46 ± 33.90 | NR | 13.77 ± 6.17 | NR |
| Beaumont 2015 (IMT during PR) | 3 weeks | IMT + standard pulmonary rehabilitation | 16 | 42 ±14 | NR | 45 ±10 | 395 ±136 | NR | NR | NR |
| Beaumont 2015 (IMT during PR) | 3 weeks | Standard pulmonary rehabilitation (no IMT) | 18 | 52 ±19 | NR | 49 ±10 | 439±105 | NR | NR | NR |
| Schultz 2018 (3-week PR + IMT) | 3 weeks | Intervention group | 300 | 51.0±15.3 | 3.22±0.91 | NR | 425.2±113.7 | NR | NR | NR |
| Schultz 2018 (3-week PR + IMT) | 3 weeks | Control group | 302 | 49.5±15.0 | 3.18±0.91 | NR | 420.1±115.1 | NR | NR | NR |

* FEV1% = FEV1 percent predicted (reported in the original article and used here as a surrogate for FEV1/FVC where applicable).
NR, Not reported in the original trial

Supplementary Table S2. GRADE evidence profile for main outcomes (reasons for downgrading are provided below the table).

| **Outcome** | **No. of studies** | **Study design** | **Risk of bias** | **Inconsistency** | **Indirectness** | **Imprecision** | **Publication bias** | **Overall certainty (GRADE)** |
| --- | --- | --- | --- | --- | --- | --- | --- | --- |
| SGRQ total score | 3 | Randomized controlled trials | Serious | Not serious | Not serious | Not serious | Undetected | **Moderate** |
| mMRC dyspnoea score | 3 | Randomized controlled trials | Serious | Not serious | Not serious | Not serious | Undetected | **Moderate** |
| FEV₁ (L) | 4 | Randomized controlled trials | Serious | Serious | Not serious | Not serious | Undetected | **Low** |
| FVC (L) | 3 | Randomized controlled trials | Serious | Serious | Not serious | Not serious | Undetected | **Low** |
| FEV₁/FVC (%) | 4 | Randomized controlled trials | Serious | Serious | Not serious | Not serious | Undetected | **Low** |
| 6MWD (m) | 6 | Randomized controlled trials | Serious | Serious | Not serious | Serious | Undetected | **Very low** |
| CAT score | 3 | Randomized controlled trials | Serious | Serious | Serious | Serious | Undetected | **Very low** |

Downgrading rationale (GRADE) for Supplementary Table S2: SGRQ total score and mMRC dyspnoea were downgraded one level for serious risk of bias. FEV1, FVC and FEV1/FVC were downgraded one level for serious risk of bias and one level for serious inconsistency. 6MWD was downgraded for serious risk of bias, serious inconsistency and serious imprecision (overall very low certainty). CAT was downgraded for serious risk of bias, serious inconsistency, serious indirectness and serious imprecision (overall very low certainty). Publication bias was not assessed because fewer than 10 studies were available per outcome.
